# Telehealth competencies for allied health professionals: A scoping review

**DOI:** 10.1177/1357633X231201877

**Published:** 2023-10-03

**Authors:** Krithika Anil, Adam R Bird, Kate Bridgman, Shane Erickson, Jennifer Freeman, Carol McKinstry, Christie Robinson, Sally Abey

**Affiliations:** 1 School of Health Professions, 6633 University of Plymouth, UK; 2 110570 School of Allied Health, Human Services & Sport, 2080 La Trobe University, Melbourne, VIC, Australia

**Keywords:** Telehealth, remote consultation, education, training, competencies, allied health

## Abstract

**Introduction:**

Telehealth has become one of the main methods of delivering allied health professional services world-wide, yet many professionals do not have sufficient training to deliver high-quality telehealth services. This review aims to identify what competencies allied health professionals require for effective telehealth service delivery.

**Methods:**

This scoping review used the Population Concept Context framework and searched the following databases: MEDLINE, CINAHL, PsychInfo, Cochrane, EMBASE, Web of Science, PEDro, United Kingdom Health Forum, WHO, Health Education England, and all UK and Australian AHP professional bodies.

**Results:**

A total of 37 articles were included out of 92,525 identified by the literature search. Competencies were related to two areas: (1) delivery of the telehealth consultation and (2) service management of telehealth consultations. The first area included the following competency themes: clinical reasoning, communication, effectively using technology, person-centred care, practice-based assessment and intervention knowledge/behaviour/skills, privacy, security, and patient safety, professionalism, and setting up the technical environment. The second area included the following competency themes: digital infrastructure, informing practice, and management. Although findings emphasised the importance of telehealth competencies, none have been implemented within education. One-third of the articles were from the discipline of psychology.

**Conclusion:**

This is the first scoping review to combine telehealth competencies reported across allied health disciplines. Although there were a vast range of competencies, they need implementation into teaching and learning to be practically useful. Most competencies were from psychology, but potentially applicable for other allied health professionals. A shared and adaptable standard for telehealth competencies would be useful to ensure high-quality practice across all allied health professionals.

## Introduction

Telehealth has been an established service delivery method for allied health professionals (AHPs) for decades,^1^ but the COVID-19 pandemic has seen a steep rise in its application in response to service delivery challenges.^1–3^ The term ‘telehealth’ refers to the delivery of healthcare services using information and communication technologies for diagnosis, treatment and prevention of disease and injuries, research, and evaluation.^4^ Telehealth is also referred to as telepractice, teledelivery, telerehabilitation, or included in broader terms such as eHealth or mHealth.

The broad benefits of telehealth are well-documented^5^ and include increased service availability, convenience, improved access to services for people living in rural and remote areas,^6^ and removal of travel and geographical barriers.^7,8^ With more service-users having had the opportunity to experience telehealth, they are more aware and potentially receptive to engaging in this service delivery method. It is likely that many AHPs will continue to offer telehealth as part of their routine practice, alongside and integrated with in-person services. The adoption of telehealth by AHPs, and the breadth of telehealth services, is predicted to continue evolving in the coming years, particularly as new health technologies emerge.

Significant investments have been made by healthcare providers and governments worldwide to facilitate the use of telehealth technologies.^9,10^ At local levels, this has included investments in infrastructure to deliver telehealth including telecommunications and videoconferencing equipment, access to videoconferencing platforms, the adaption of standardised assessments and treatment resources, and the provision of internet access with sufficient bandwidth. At a government level, significant investments have been made in a wide scale, secure telehealth video-consulting platforms, upgrades to technological infrastructure, and funding to subsidise some services. Further investments will continue to be required to meet the future needs associated with new telehealth technologies and services.^11^ This is emphasised by Thomas et al.,^12^ who stated that telehealth implementation demands a clear strategy that includes the determination of roles and responsibilities across the organisation. This cannot be achieved if the AHP workforce does not possess the required skills, knowledge, and behaviours to provide safe, efficient, and effective telehealth services.

For many AHPs, the uptake of telehealth as a service delivery method was a necessity due to the social distancing requirements and practice restrictions associated with COVID-19. Many had limited or no prior experience using telehealth (e.g. Buckingham et al^13^ and Hall-Mills et al^14^) and hence had not considered or acquired the competencies required, leading to feelings of uncertainty, fear, and apprehension (e.g. Erickson et al^15^). To deliver safe and effective telehealth services, AHPs require additional skills and behaviours to those required for in-person consultations.

In addition to AHPs providing telehealth services, there is an ongoing need to educate healthcare students in the use of telehealth. Bridgman et al.^16^ synthesised the literature relating to the perspectives of allied health students on clinical placements that incorporated telehealth. While little has been published on this topic, a key finding was that considerable work is needed to adequately prepare students for using telehealth. Historically, preprofessional or university education has focussed on service implementation relating to technology, legal issues, and developing the necessary policies, education, and training for telehealth in an explicit, systematic manner.^17^

Several competency frameworks describing core capabilities for telehealth service delivery have been published to support AHPs. A Delphi study, using a panel of 40 AHPs, developed, reviewed, and ratified lists of telehealth competencies: Tack^18^ published the ‘AHP Digital Competency Framework’, developed using a three-round Delphi study to gain agreement upon 124 competencies within 10 domains. This publication was not peer-reviewed and there is limited detail about the methodology that was undertaken. The framework broadly covers digital health competencies, rather than a specific focus on telehealth. Davies et al.^19^ also utilised a three-round Delphi design with 130 international stakeholders to publish a peer-reviewed framework of the core capabilities physiotherapists require to provide services using videoconferencing. In contrast to Tack's^18^ framework, a detailed description of the methodology was provided to support their core capability framework. Expert consensus has also guided the development of telehealth competency frameworks for nurses,^20^ medical professionals,^21^ and interprofessional telebehavioural health competencies.^22^

AHP associations have published position statements and guidelines outlining the standards of care necessary to deliver telehealth services aligning their professional regulations and policies. For example, guidelines for the practice of telepsychology have been published by the American Psychological Association,^23^ while Speech Pathology Australia published a position statement, informed by a broad working party of experienced speech pathologists and researchers, to assist speech pathologists implementing telehealth in the Australian context.

To date, no publication has sought to synthesise the existing telehealth competencies for AHPs developed by expert panels and professional, national, or international organisations. While frameworks, guidelines and position statements provide guidance regarding telehealth core capabilities, no study has sought to incorporate the telehealth competencies reported across all published research and the grey literature, including opinion articles, guidance documents, and non-systematic literature reviews.

## Review aim and objectives

This scoping review aimed to answer the following question: Which competencies do AHPs require for effective telehealth service provision? The objectives were to identify:

1. Existing telehealth competencies for AHPs developed by professional, national, or international institutes.
2. Telehealth competencies and current practices delivered through education programmes and/or practice placements for AHPs.

## Methods

The review question was framed according to the Population Concept Context (PCC) framework^24^ and registered with the Open Science Framework (https://osf.io/vrp62).

### Inclusion criteria

#### Population

This review considered studies involving at least one AHP field. The included AHPs listed in Table 1 were derived from two United Kingdom-based sources (i.e. the Health and Care Professions Council^25^ and National Health Service (NHS) England^26^) and two Australian-based sources (i.e. Allied Health Professions Australia^27^ and the Australian Government Department of Health^28^). Professions related to nursing, medicine, and dentistry were not included.

**Table 1.** Allied health professions are included in this scoping review.

Arts therapy	Operating department practitioners
Audiology	Optometry
Biomedical scientists	Orthoptists
Chiropractic	Orthotics/prosthetics
Chinese medicine practitioners	Osteopathy
Clinical scientists	Paramedic practitioners
Credentialled diabetes educators	Pedorthist
Dietetics	Perfusion
Diversional therapists	Pharmacy
Drama therapists	Physiotherapy
Exercise physiology	Podiatry
Genetic counselling	Psychology
Hearing aid dispensers	Rehabilitation counselling
Medical radiations/radiographers	Social work
Music therapy	Sonography
Occupational therapy	Speech pathology

#### Concept

Articles that reported telehealth competencies, including those in pre-registration education programmes, for AHPs, were included in this review. Technology incorporating asynchronous communication, such as emails or mobile apps, was not considered. Technology must have included synchronous communication (with or without an asynchronous element) to be considered. Competencies were broadly defined as the knowledge, skills, and behaviours needed to deliver services of an AHP efficiently and professionally.^29^

#### Context

This review considered educational and practice-based environments, including both pre and post-registration contexts.

#### Types of literature

All study types and grey literature, including opinion articles, guidance documents, and non-systematic literature reviews were included. Theses and books were excluded. The review implemented a date restriction from 2012 to August 2022, because literature relating to technologies before 2012 were considered out of date due to the rapid speed of technology development.^30,31^

### Search strategy

A three-step search strategy was utilized. An initial limited search of MEDLINE and AHP professional body websites was undertaken to estimate the volume of relevant literature and to identify search terms. A second search using the developed search terms was undertaken and adapted across each of the following databases: MEDLINE, CINAHL, PsychInfo, Cochrane, EMBASE, Web of Science, PEDro, United Kingdom Health Forum, WHO, Health Education England, and all UK and Australian professional bodies for AHPs. The third strategy involved searching for additional studies within the reference list of all studies that met the inclusion criteria. Please see the Supplemental Material titled ‘Record of Online Searches’ for search strategy and terms.

### Study selection

Following the search, all identified references were imported into EndNote (software allowing the easy organisation of study references, including study titles and abstracts^32^). After the removal of duplications, references were uploaded to the online Rayyan tool, an organisational tool for systematic or scoping reviews.^33^ Titles and abstracts were divided equally amongst each member of the review team (i.e. all co-authors) and screened independently for assessment against the inclusion criteria. Our team includes backgrounds from multiple AHPs: physiotherapy, podiatry, speech-language pathology, and occupational therapy, which ensured article selection and analysis were not from the perspective of a single profession.

An agreement check was conducted at the conclusion of the abstract screening, where each team member was paired and checked 10% of each other's screening. To ensure that cultural understanding was aligned, a UK team member was paired up with a team member from Australia. Any conflicts were resolved through discussions with the team.

**Figure 1.**
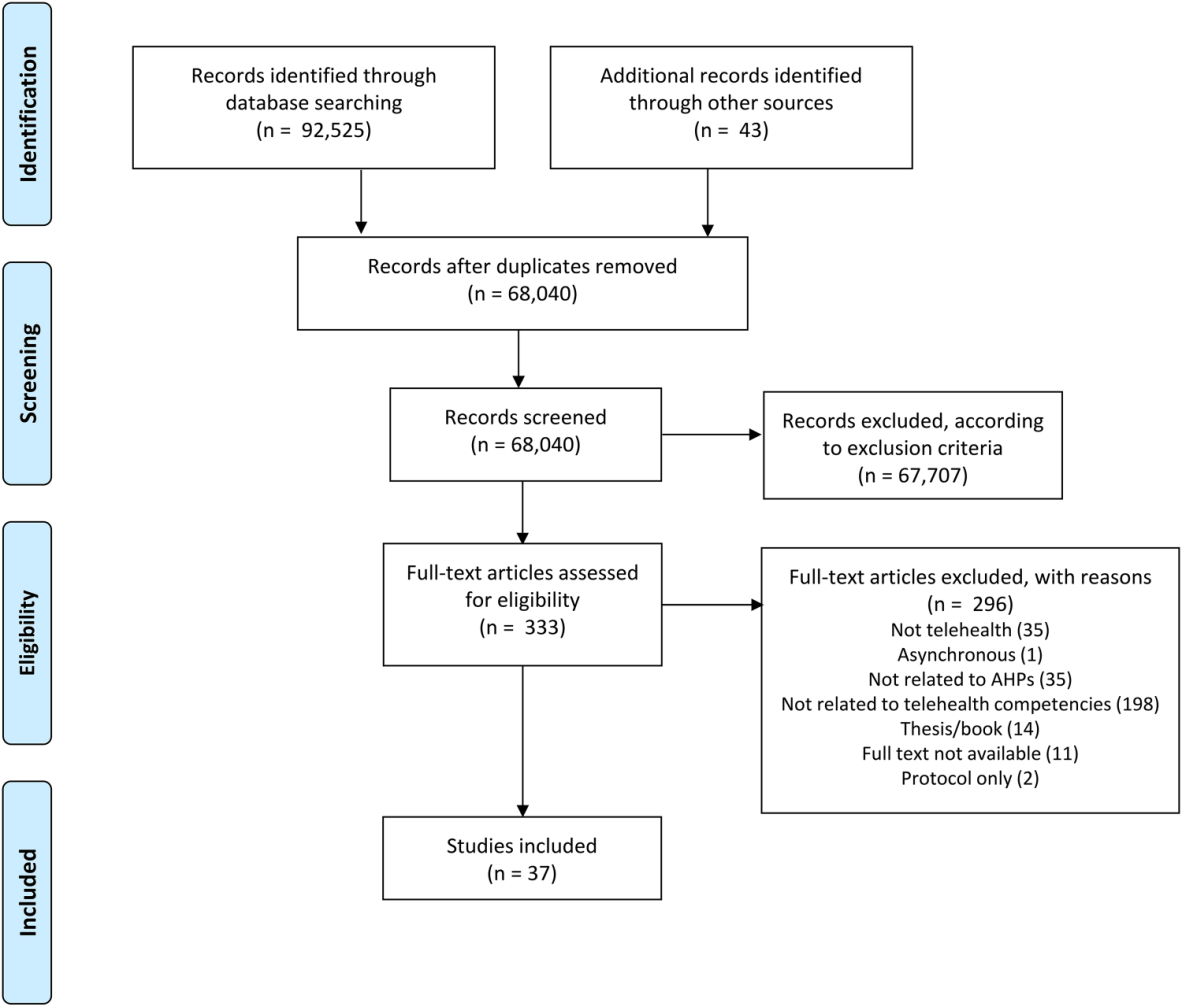
Preferred Reporting Items for Systematic Reviews and Meta-Analyses (PRISMA) flow diagram that charts the study identification process.

The full texts of potentially eligible studies were retrieved and assessed in detail against the inclusion criteria by the review team. Full-text studies that did not meet the inclusion criteria were excluded and were displayed in a Preferred Reporting Items for Systematic Reviews and Meta-Analyses (PRISMA) flowchart (Figure 1). Any disagreements between reviewers were resolved through discussion within the review team.

### Data extraction and synthesis

Data extraction of included studies were conducted by the review team. Narrative data extraction were conducted using an Excel spreadsheet according to the objectives of this review. Any disagreements between reviewers were resolved through discussion. The narrative synthesis of the findings was structured according to the review objectives. The findings are presented narratively in the results section aided by appropriate tables and figures.

## Results

Thirty-seven articles were included in this scoping review (Figure 1; see Table 2 for article characteristics). Five were original research studies,^34–38^ four were Delphi studies,^18,19,39,40^ and two were systematic/scoping review studies.^41,42^ The remaining 26 articles were guideline documents (n = 10),^43–52^ opinion pieces (n = 9),^53–61^ non-research reports (n = 2),^62,63^ policy/standards (n = 3),^64–66^ and a non-systematic literature review (n = 1).^67^ The five research studies involved the following study designs: cross-sectional (n = 2),^37,38^ cohort (n = 1),^34^ qualitative (n = 1),^36^ and observational with only one condition (n = 1).^35^ Twenty-three articles were from the USA,^34–38,42,45,47,50,51,53–61,63,65,67,68^ while the remaining articles were from Australia (n = 6),^19,43,46,49,64,66^ the UK (n = 4),^18,41,48,62^ Saudi Arabia (n = 2),^39,44^ Canada (n = 1),^40^ and world-wide (n = 1).^52^

**Table 2.** Characteristics of the included articles.

Authors (date)	Country Origin	Aim	Article type	Profession
Allied Health Professions Australia (2018)^43^	Australia	To provide AHPs with practical advice on how to conduct telehealth consultations, and develop a better understanding of telehealth	Guidance	General AHPs
Almubark et al. (2022)^44^	Saudi Arabia	To provide a specialty-specific telehealth practice guide for rehabilitation practitioners developed by an expert panel in the field of rehabilitation	Guidance	Occupational therapy, physiotherapy, and speech pathology
Alqahtani et al. (2021)^39^	Saudi Arabia	To provide a guide to the implementation of telepsychology in the context of the COVID-19 pandemic and to extend its implementation for use fundamentally as the main guideline for telepsychology services in Saudi Arabia and other Arabic communities	Delphi study	Psychology
American Psychology Association (2013)^45^	USA	To address the developing area of psychological service provision commonly known as telepsychology	Guidance	Psychology
Andrews et al. (2021)^41^	UK	To explore the subject of telephone reviews and how therapeutic review radiographers might need to adapt communication skills so that they can continue to effectively assess and manage radiotherapy treatment reactions remotely	Systematic/scoping review	Medical radiations/radiographers
Audiology Australia (2022)^46^	Australia	To support the delivery of hearing services safely and effectively through tele-audiology, and enhance access to high-quality hearing care across Australia	Guidance	Audiology
Badowski et al. (2021)^53^	USA	To educate clinical pharmacists on available telehealth technologies and summarize methods for training current and prospective clinical practitioners to practice in telehealth environments	Opinion	Pharmacy
Badowski et al. (2018)^54^	USA	To provide direction for clinical pharmacists providing telehealth	Opinion	Pharmacy
Barnett and Kolmes (2016)^55^	USA	To outline the ethical, legal, and clinical issues for practitioners regarding tele-mental health practice	Opinion	Psychology
Baumes et al. (2020)^56^	USA	To cross-examine the ethical codes and guidelines of different, but related fields of practice and to discuss potential implications for telehealth-based applied behaviour analysis service delivery	Opinion	Psychology
Bautista et al. (2020)^34^	USA	To describe the development, implementation, and evaluation of an interprofessional rotation for third-year pharmacy and medical students focused on telehealth outreach to service-users at high risk for delaying care	Research study	Pharmacy
Bilder et al. (2020)^47^	USA	To provide rapid guidance about tele-neuropsychology in response to the COVID-19 pandemic	Guidance	Psychology
Bishop et al. (2013)^62^	UK	To describe the PhysioDirect system used in the trial and how physiotherapists were trained and supported to use the system and deliver the PhysioDirect service	Report (non-research)	Physiotherapy
Cason et al. (2013)^57^	USA	To provide the current position of the American Occupational Therapy Association regarding the use of telehealth by occupational therapists and occupational therapy assistants to provide occupational therapy services	Opinion	Occupational therapy
Cason et al. (2018)^58^	USA	To state the current position of the American Occupational Therapy Association regarding the use of telehealth by occupational therapy practitioners	Opinion	Occupational therapy
Cheung et al. (2021)^40^	Canada	To develop consensus-based statements to guide the optimal provision of virtual care for clinicians caring for service-users with cancer	Delphi study	Pharmacy and medical radiations/radiographers
Cohn (2012)^59^	USA	To provide an overview of tele-ethics, the ethical delivery of clinical services via the internet and telecommunication technologies by speech-language pathologists and audiologists	Opinion	Audiology and speech pathology
Cooper et al. (2019)^68^	USA	To identify the natural integration of the pillars of counselling psychology with the major domains of telepractice and to link telepractice to the values and mission of counselling psychologists	Opinion	Psychology
Crockett et al. (2020)^63^	USA	To describe the impact, changes, and outcomes achieved by a large, multifaceted applied behavioural analysis clinical programme	Report (non-research)	Psychology
Davies et al. (2021)^19^	Australia	To develop a core capability framework for physiotherapists to deliver quality care via videoconferencing, using an international consensus process involving consumers, physiotherapy clinicians and researchers, and representatives of private health insurers and physiotherapy professional organisations	Delphi study	Physiotherapy
Digital Health Skills (2019)^48^	UK	To provide digital competencies that are applicable to all applied psychologists and psychological practitioners working online and via telephone	Guidance	Psychology
Edwards-Stewart et al. (2019)^60^	USA	To identify best practices for incorporating apps into treatment, including competence in the use of smartphones in general and familiarity with the specific apps recommended	Opinion	Psychology
Eikelboom et al. (2021)^49^	Australia	To collate information from the literature on current research and practice evidence for tele-audiology, integrated with stakeholder views collected via surveys and interviews	Guidance	Audiology
Exercise and Sports Science Australia (2020)^64^	Australia	To describe the core elements of effective and ethical clinical exercise physiology practice	Policy/standard	Exercise physiology
Gifford et al. (2012)^35^	USA	To describe one model for delivering behavioural telehealth training of competencies and evaluate its effectiveness in developing those competencies	Research study	Psychology and social work
Henry et al. (2017)^42^	USA	To describe interpersonal clinician behaviours and attributes of care in telehealth delivery related to provider/service-user interaction	Systematic/scoping review	Psychology
Luxton et al. (2014)^67^	USA	To discuss the specific factors that influence the validity and reliability of remote psychological assessments and to provide best practice recommendations	Non-systematic review	Psychology
Maheu et al. (2021)^61^	USA	To present a telebehavioural health competency framework that assists in using technology to afﬁrm and adapt existing in-person clinical psychology practices	Opinion	Psychology
Myers (2017)^65^	USA	To provide a clinical guideline for the delivery of child and adolescent mental health and behavioural services by a licensed healthcare provider through real-time videoconferencing	Policy/standard	General AHPs
Optometry Australia (2021)^66^	Australia	To provide a telehealth clinical practice guide for Australian optometrists	Policy/standard	Optometry
Overby (2018)^36^	USA	To qualitatively examine the perceptions of speech–language pathology/therapy faculty, students, and clinicians to ascertain effective pedagogical approaches for telepractice service delivery	Research study	Speech pathology
Overby and Baft-Neff (2017)^37^	USA	To quantitatively examine the perceptions of speech-language pathology/therapy faculty, graduate students, and telehealth clinicians regarding telepractice service delivery	Research study	Speech pathology
Perle (2022)^38^	USA	To evaluate mental health providers’ telehealth educational activities both prior to and following implementation	Research study	Psychology and social work
Scott et al. (2022)^50^	USA	To provide ethical considerations and suggestions for potential practitioners of tele-neuropsychology based on new models of practice derived in response to the COVID-19 pandemic	Guidance	Psychology
Snodgrass et al. (2017)^51^	USA	To describe a framework of parent training and coaching that can be used to incorporate speech-language therapy strategies that support parents during home-based activities with their children	Guidance	Speech pathology
Tack (2020)^18^	UK	To provide recommendations to guide the implementation of innovative technology in practice and facilitate the development of the healthcare workforce	Delphi study	General AHPs
World Federation of Occupational Therapists (2014)^52^	Global	To state the World Federation of Occupational Therapists’ position on the use of telehealth for the delivery of occupational therapy services	Guidance	Occupational therapy

AHPs: Allied Health Professionals; USA: United States of America; UK: United Kingdom.

### Professions representation

Ten professions were represented within the included articles: psychology,^35,38,39,42,45,47,48,50,55,56,60,61,63,67,68^ occupational therapy,^44,52,57,58^ pharmacy,^34,40,53,54^ social work,^35,38,61^ speech pathology,^36,37,44,51,59^ audiology,^46,49,59^ physiotherapy,^19,44,62^ medical radiations/radiographers,^40,41^ exercise physiology,^64^ and optometry.^66^ Table 3 shows the number of articles that represented these professions, including those articles involving multiple professions.

**Table 3.** The number of articles that represented the professions identified within the included articles.

Profession	Number of articles
Psychology	12
Occupational therapy	3
Pharmacy	3
Psychology and social work	3
General AHPs	3
Speech pathology	3
Audiology	2
Physiotherapy	2
Occupational therapy, physiotherapy, and speech pathology	1
Medical radiations/radiographers	1
Pharmacy and medical radiations/radiographers	1
Audiology and speech pathology	1
Exercise physiology	1
Optometry	1

### Competency themes

The competency themes listed in Tables 4 and 5 were derived from our interpretation of the information within the included articles. The term ‘competency’ was not a universal term and was not consistently used within the literature. Therefore, the resulting competencies reported here should be considered as a consolidation of telehealth knowledge, skills, and behaviours from the included articles rather than definite competencies. The competencies extracted and synthesised from the literature only include those deemed to differ from those of in-person delivery. Therefore, these competencies should not be considered in isolation, but within the context of existing standards of practice, service delivery guidelines, and governance relevant to the profession and setting.

**Table 4.** Competency themes regarding the delivery of telehealth consultations.

**DELIVERY OF TELEHEALTH CONSULTATIONS**
**Clinical reasoning**
Check whether a telehealth consultation is an appropriate medium based upon the service-user's health condition^38,56,57^
Know how to assess during a telehealth consultation whether in-person is required instead^43,48,54–56,59,62^
Know how to support service-users who have not previously used remote technologies^47,64^
Know whether telehealth is an appropriate medium to communicate updates to service-users^40^

**Communication**
**General communication**
Know how to develop a contingency plan in case of technical failures and communicate this with the service-user^35,43^
Know how to work with the service-user to develop a communication plan during and outside of telehealth consultations as needed^46^
**Pre-consultation communication**
Ensure there is a plan to resolve the issue of becoming disconnected from the service-user^63^
Explain the implications and limitations of telehealth versus in-person to the service-user when offering a telehealth service^49,57^
Know how to navigate failed communications, such as miscommunication, equipment failure, or service-user's loss of faith in the telehealth service^49^
Make the service-user aware of their rights and responsibilities when receiving a telehealth consultation, including their right to refuse telehealth^65^
Prepare the client for the telehealth consultation by providing relevant and explicit information about the timing, technology, and instructions^43^
**During consultation communication**
Be proactive in ensuring the client understands the telehealth services being offered and understanding their service preferences^46,53,58,66^
Know how to address non-verbal cues when using telehealth^35,46,47,55,61,63,67^
Know how to effectively communicate via telephone as needed for a telehealth consultation^41,62^
Know how to effectively teach the service-user the basics of technology used for the telehealth consultation^36,47^
Know how to explicitly introduce yourself and check the client's identity^40,43^
Know how to proactively and thoughtfully engage with the client using telehealth^36,40–42,46,48,53,54,62,64^
**Post-consultation communication**
Document any changes to assessments that were adapted for telehealth^58,59^

**Effectively using technology**
Ensure you are familiar with the technology and systems used for telehealth for the smooth running of the consultation and any troubleshooting^38,45,55,57,58,60,68^
Know how to collaborate effectively with service-users using relevant digital technologies^36,48^
Know how to test and troubleshoot your digital equipment and the service-users’ equipment^43,46,67^
Understand how your speech may be distorted through communication technologies^46^

**Person-centred care**
Be aware of how inadequate acceptance of telehealth by the service-user may negatively affect the validity and reliability of any remote assessments^67^
Ensure that the service-user provides informed consent on their digital data management, and any recording or sharing of any of their telehealth consultation^40,43,56,59,60^
Explicitly check that the service-user provides informed consent to receiving a telehealth consultation as acceptable^43,57,59^

**Practice-based assessment and intervention KBSs**
Know how to choose the most appropriate technology for specific telehealth consultation contexts^36,38,44,47,48,57–59,64^

**Privacy, security, and safety**
Check that it is safe to conduct any telehealth assessments^42,43,49,59^
Provide explicit information on how the service-user's digital data is managed, and how their privacy and confidentiality will be protected^35,39,43,46,52,60,64,68^
Provide information about any other persons that will be at any of the planned telehealth consultations^43,46,54,56,59^
Understand the terms and conditions of any software used in telehealth consultation to ensure data is kept confidential^60^

**Professionalism**
Have a professional environment and reduce clutter and other distractions from the background ^59,63^
Keep updated on tax-related information for telehealth services ^43^
Understand how to ensure the safety of service-users when engaging in telehealth ^43,59^
Understand the limits of own competence when translating in-person service to telehealth ^38,48,55,68^

**Setting up the technical environment**
Be aware of how sound and background noise can be measured accurately via telehealth compared to in-person ^46^
Know how to efficiently set up technologies in preparation for any telehealth consultation ^38^
Know how to regularly test your technology to ensure that it works appropriately and safely ^35,47,49^

**Table 5.** Competency themes regarding the management of telehealth services.

**TELEHEALTH SERVICE MANAGEMENT**
**Digital infrastructure**
Be aware of security and confidentiality risks associated with digital infrastructure^18^
Have a billing system that incorporates telehealth consultations and associated billing codes^54^
Have a central electronic health record with an understanding of the quality, impact, and use of data in practice^38,40,54^
Have an understanding of business systems and data related to telehealth service^38,43,53^
Understand the local digital infrastructure, policies, guidelines, and frameworks^35,40,43,46^

**Informing practice**
Keep up to date with relevant professional body best telehealth practices, research, and resources^19,38,39,42,43,45,46,49,52,55,57,59,61,65,67^
Know how to reflect on digital practices^43,48^
Know how to share and promote telehealth practice^37,48,62^
Understand how the differences in culture and social background can impact telehealth^34,35,37,38,47,48,50,52,54–57,59,63,67,68^
Understand how to implement best practices to ensure privacy and confidentiality for the service-user and others involved in the telehealth service^19,36,38,40,43,45–47,49–52,54–57,59,60,64,67^
Understand your local and national laws and scope of practice when engaging in telehealth^37,38,43,45–47,54,56–59,61,65^

**Management**
Ensure to have a mechanism in place where service-users are aware of their rights of accessing telehealth services including communication for complaints and grievances^49^
Ensure to maintain online professional boundaries^48,59,68^
Have a standard process for documentation^54^
Minimise barriers to accessibility of telehealth services for all service-user groups and providers^40,43^
Understand how to collaborate inter-professionally and with local providers to improve any telehealth service^34,36,38,40,42,43,46,52–54^
Understand how to plan and manage the delivery of telehealth services including contingencies and episodes of care^19,35,43,46,53,59^
Understand how to set up remote supervision of students or trainees^46^

Table 4 presents competency themes relating to the delivery of telehealth consultations. This includes eight overarching themes:

- Clinical reasoning
- Communication
- Effectively using technology
- Person-centred care
- Practice-based assessments and intervention KSBs
- Privacy, security, and safety
- Professionalism
- Setting up the technology

In addition to the competencies related to the delivery of telehealth consultations, the literature highlighted specific considerations related to telehealth service management. Whilst the previously outlined competencies apply to the individual AHPs delivering the telehealth consultation, the service delivery competencies are relevant to those responsible for the overall management and delivery of AHP telehealth services within an organisation. These included three overarching themes:

- Digital infrastructure
- Informing practice
- Management

Explanation of how the competencies were developed was not reported in 16 of the included articles. Of the remaining 24 articles, five developed competencies through a Delphi process,^39,40,61,65^ seven were developed through a non-Delphi expert critical evaluation,^34–36,44,45,47,62^ five were based on existing standards or guidance,^46,56,59,65,68^ and seven were based on a literature review.^19,37,38,41,42,49,54^

Two relevant gaps in research were reported by three of the included articles. Two articles stated that the effectiveness of assessments should be examined by comparing those delivered via telehealth and in-person services.^38,40^ One article highlighted the need to compare the differences in specific ethical considerations between using telehealth and in-person services.^50^

Recommendations were suggested to support the implementation of telehealth competencies. Bishop et al.^62^ recommended that the delivery of telehealth should be shared between all staff to avoid a few staff members taking on the majority of telehealth delivery within a service. Eikelboom et al.^49^ suggested that a facilitator-led model of telehealth delivery will benefit the coordination of clinical procedures that cannot be delivered remotely. Finally, Alqahtani et al.^39^ suggested the implementation of a multi-professional team to efficiently run a telehealth service that addresses a diverse range of health or social care issues.

## Discussion

This is the first scoping review to combine telehealth competencies reported across Allied Health disciplines. This is significant considering telehealth is a universal service delivery modality that has the potential to span multiple disciplines within and between health services. The 40 articles included in this scoping review revealed that telehealth competencies have been considered by 10 Allied Health disciplines over the past decade. More than 77.5% of the yield was published pre-COVID-19, indicating that interest in telehealth competencies predates the pandemic. Almost one-third of publications were from the discipline of psychology which is largely communication-based. Competencies for disciplines whose typical practice requires equipment, and hands-on assessment and management (e.g. physiotherapy, exercise physiology, podiatry, and occupational therapy) may require specific additional competencies that were not reflected in this review. Further, while 10 of the 32 disciplines are represented in this review, it is unclear whether the remaining two-thirds do not engage in telehealth, or simply have no published guidelines or studies.

Eight articles specifically presented competencies within a standard or framework, yet five of these articles omitted most of the competencies identified in this review. This could be reflective of inconsistent agreement about individual or discrete telehealth competencies, or it could be due to the variance in the use of telehealth across individual allied health disciplines. One article^48^ omitted details of how the competencies were developed. The two remaining articles, by Davies et al.^19^ relating to physiotherapy and Tack^18^ relating to AHPs generally, covered most of the competencies identified in this review. However, as mentioned in the introduction, the Tack^18^ framework was not peer-reviewed and includes a broad range of competencies related to digital health rather than the delivery of telehealth specifically. Davies et al.^19^ conducted a Delphi study published in a peer-reviewed journal with a detailed description of a robust methodology and with a focus on telehealth yet is specific to physiotherapy only.

The competencies distilled from this scoping review include eight domains relating to the delivery of telehealth consultations and three domains relating to telehealth service management. The clinical competencies relating to clinical reasoning, communication, person-centred care, and professionalism appear to be more recently reported telehealth competencies when compared to early telehealth research that focussed on implementation logistics, legislation, and insurance. The inclusion of practice-based assessment and intervention also reflects more closely the main purpose of telehealth. These competencies are however largely drawn from frameworks, guidelines, and expert opinion, with only 12.5% of the yield relating to original research. This is important when considering whether telehealth competencies frameworks have been validated rather than theoretically considered to be evidence-based practice. None of the standards or frameworks identified in this scoping review have been validated. Thus, they are limited to expert opinion rather than supported by empirical evidence.

The second objective of this review was to determine any literature relating to education programmes. Only five articles (all research studies) evaluated telehealth education and training, whereas the remaining articles related to telehealth position statements, and guidelines, however, these documents do not consider how these skills, behaviours, and knowledge will be imparted to current and future AHPs. This is especially important to evaluate as it will inform best practices in implementing a telehealth service efficiently and sustainably. Cook et al.^69^ found that negative assumptions about the ease of telehealth implementation often resulted in the decision to not offer telehealth. Understanding how best to train practitioners in telehealth may relieve these negative assumptions and showcase how telehealth can complement existing health and social care services.

Finally, the term *competency* is not a universal term commonly used in the literature. Instead, terms relating to knowledge, skills, and behaviour are reported. Interestingly, many articles did not explicitly differentiate between competencies exclusively required for telehealth consultations compared to in-person consultations. While this is likely to ensure that generic competencies are not omitted or assumed for telehealth delivery, it may confuse what are generic or transferable skills for all service modalities (i.e. strong inter-personal skills and active listening) compared to telehealth-specific skills (i.e. be proficient in the technology platform being used). The further delineation between generic or transferrable skills and telehealth-specific competencies would assist education relating to effective telehealth delivery by pre-registration and practising AHPs.

This scoping review has several limitations. Although this review conducted an extensive search within grey literature sources, it is possible that there are more telehealth competency documents that are likely unpublished and may have been missed. The search strategy was biased towards the English language, where non-English relevant articles have likely been missed. This review also included a date restriction, where no articles prior to 2012 were included in the literature search in order to exclude outdated information, where this may have also excluded some relevant documents.

This review highlights the need to explicitly define the competencies needed for telehealth delivery and evaluate them in terms of education and training. Future work must examine the effectiveness of such telehealth education and training in order to prepare our health and social care workforce to delivery efficient, high-quality telehealth services.

## Supplemental Material

sj-docx-1-jtt-10.1177_1357633X231201877 - Supplemental material for Telehealth competencies for allied health professionals: A scoping review Supplemental material, sj-docx-1-jtt-10.1177_1357633X231201877 for Telehealth competencies for allied health professionals: A scoping review by Krithika Anil, Adam R Bird, Kate Bridgman, Shane Erickson, Jennifer Freeman, Carol McKinstry, Christie Robinson and Sally Abey in Journal of Telemedicine and Telecare
