# Supplementary material for: Telehealth competencies for allied health professionals: A scoping review
Source: J Telemed Telecare. 2023 Oct 3;31(4):487–99. doi: 10.1177/1357633X231201877 (PMC12044212; doi:10.1177/1357633X231201877)
Supplement: sj-docx-1-jtt-10.1177_1357633X231201877 - Supplemental material for Telehealth competencies for allied health professionals: A scoping review [file sj-docx-1-jtt-10.1177_1357633X231201877.docx]

**Supplementary Materials**

**Record of Online Searches**

The below search strategy uses a logic grid and is based on the PCC framework. The searches were recorded and provided as an appendix with dates for any publications of the completed review.

| *Population* | *Concept 1* | *Concept 2* | *Context* |
| --- | --- | --- | --- |
| Art therap* | Tele* | Competenc* | Teach* |
| Audiolog* | Phone | Standard* | Learn* |
| Biomedical scien* | Video | Knowledge | Curriculum |
| Chiropract* | Remote | Skill* | Prac* |
| Chinese medic* | Simulat* | Behavio* | Course* |
| Clinical scien* |  |  | Prog* |
| Diabetes educat* |  |  | Module |
| Dietetics |  |  | Apprenticeship |
| Dietitian |  |  | Professional development |
| Diversional therap* |  |  |  |
| Drama therap* |  |  |  |
| Exercise scien* |  |  |  |
| Exercise physio* |  |  |  |
| Genetic counsel* |  |  |  |
| Hearing aid dispens* |  |  |  |
| Radiation oncology medical physics |  |  |  |
| Radiation therap* |  |  |  |
| Radiograph* |  |  |  |
| Music therap* |  |  |  |
| Occupational therap* |  |  |  |
| Operating department pract* |  |  |  |
| Optometr* |  |  |  |
| Orthopti* |  |  |  |
| Orthoti* |  |  |  |
| Prostheti* |  |  |  |
| Osteopath* |  |  |  |
| Paramedic* |  |  |  |
| Pedorthist |  |  |  |
| Perfusion* |  |  |  |
| Pharmac* |  |  |  |
| Physiotherap* |  |  |  |
| Podiatr* |  |  |  |
| Psycholog* |  |  |  |
| Rehabilitation counsel* |  |  |  |
| Social work* |  |  |  |
| Sonograph* |  |  |  |
| Speech therap* |  |  |  |
| Speech language therap* |  |  |  |

1. Each column was searched individually
2. All individual searches based on each column were combined with “AND”
3. Grey literature searches were tailored according to the individual database searching tools

*Database*: MEDLINE

*Date*: 15/07/2022

| **ID** | **Search Term(s)** | **Hits** |
| --- | --- | --- |
| 1 | Art therap* OR Audiolog* OR Biomedical scien* OR Chiropract* OR Chinese medic* OR Clinical scien* OR Diabetes educat* OR Dietetics OR Dietitian OR Diversional therap* OR Drama therap* OR Exercise scien* OR Exercise physio* OR Genetic counsel* OR Hearing aid dispens* OR Radiation oncology medical physics OR Radiation therap* OR Radiograph* OR Music therap* OR Occupational therap* OR Operating department pract* OR Optometr* OR Orthopti* OR Orthoti* OR Prostheti* OR Osteopath* OR Paramedic* OR Pedorthist OR Perfusion* OR Pharmac* OR Physiotherap* OR Podiatr* OR Psycholog* OR Rehabilitation counsel* OR Social work* OR Sonograph* OR Speech therap* OR Speech language therap* | 8,897,141 |
| 2 | Competenc* OR Standard* OR Knowledge OR Skill* OR Behavio* | 5,092,408 |
| 3 | Teach* OR Learn* OR Curriculum OR Prac* OR Course* OR Prog* OR Module OR Apprenticeship OR Professional development | 6,857,007 |
| 4 | ID1 AND ID2 AND ID3 AND Tele* | 11,070 |
| 5 | ID1 AND ID2 AND ID3 AND Phone | 3,455 |
| 6 | ID1 AND ID2 AND ID3 AND Video | 7,516 |
| 7 | ID1 AND ID2 AND ID3 AND Remote | 2,728 |
| 8 | ID1 AND ID2 AND ID3 AND Simulat* | 11,849 |

Date limiter (2012) from ID 4 onwards

*Database*: CINHAL

*Date*: 15/07/2022

| **ID** | **Search Term(s)** | **Hits** |
| --- | --- | --- |
| 1 | Art therap* OR Audiolog* OR Biomedical scien* OR Chiropract* OR Chinese medic* OR Clinical scien* OR Diabetes educat* OR Dietetics OR Dietitian OR Diversional therap* OR Drama therap* OR Exercise scien* OR Exercise physio* OR Genetic counsel* OR Hearing aid dispens* OR Radiation oncology medical physics OR Radiation therap* OR Radiograph* OR Music therap* OR Occupational therap* OR Operating department pract* OR Optometr* OR Orthopti* OR Orthoti* OR Prostheti* OR Osteopath* OR Paramedic* OR Pedorthist OR Perfusion* OR Pharmac* OR Physiotherap* OR Podiatr* OR Psycholog* OR Rehabilitation counsel* OR Social work* OR Sonograph* OR Speech therap* OR Speech language therap* | 1,279,226 |
| 2 | Tele* OR Phone OR Video OR Remote OR Simulat* | 254,242 |
| 3 | Competenc* OR Standard* OR Knowledge OR Skill* OR Behavio* | 1,364,595 |
| 4 | Teach* OR Learn* OR Curriculum OR Prac* OR Course* OR Prog* OR Module OR Apprenticeship OR Professional development | 2,053,436 |
| 5 | ID1 AND ID2 AND ID3 AND ID4 | 6,030 |

Date limiter (2012) on ID 5

*Database*: PsychInfo

*Date*: 15/07/2022

| **ID** | **Search Term(s)** | **Hits** |
| --- | --- | --- |
| 1 | Art therap* OR Audiolog* OR Biomedical scien* OR Chiropract* OR Chinese medic* OR Clinical scien* OR Diabetes educat* OR Dietetics OR Dietitian OR Diversional therap* OR Drama therap* OR Exercise scien* OR Exercise physio* OR Genetic counsel* OR Hearing aid dispens* OR Radiation oncology medical physics OR Radiation therap* OR Radiograph* OR Music therap* OR Occupational therap* OR Operating department pract* OR Optometr* OR Orthopti* OR Orthoti* OR Prostheti* OR Osteopath* OR Paramedic* OR Pedorthist OR Perfusion* OR Pharmac* OR Physiotherap* OR Podiatr* OR Psycholog* OR Rehabilitation counsel* OR Social work* OR Sonograph* OR Speech therap* OR Speech language therap* | 3,442,680 |
| 2 | Competenc* OR Standard* OR Knowledge OR Skill* OR Behavio* | 2,408,237 |
| 3 | Teach* OR Learn* OR Curriculum OR Prac* OR Course* OR Prog* OR Module OR Apprenticeship OR Professional development | 2,448,304 |
| 4 | ID1 AND ID2 AND ID3 AND Tele* | 3,512 |
| 5 | ID1 AND ID2 AND ID3 AND Phone | 2,587 |
| 6 | ID1 AND ID2 AND ID3 AND Video | 8,855 |
| 7 | ID1 AND ID2 AND ID3 AND Remote | 1,690 |
| 8 | ID1 AND ID2 AND ID3 AND Simulat* | 8,557 |

Date limiter (2012) from ID 4 onwards

*Database*: Cochrane Library

*Date*: 15/07/2022

| **ID** | **Search Term(s)** | **Hits** |
| --- | --- | --- |
| 1 | Art therap* OR Audiolog* OR Biomedical scien* OR Chiropract* OR Chinese medic* OR Clinical scien* OR Diabetes educat* OR Dietetics OR Dietitian OR Diversional therap* OR Drama therap* OR Exercise scien* OR Exercise physio* OR Genetic counsel* OR Hearing aid dispens* OR Radiation oncology medical physics OR Radiation therap* OR Radiograph* OR Music therap* OR Occupational therap* OR Operating department pract* OR Optometr* OR Orthopti* OR Orthoti* OR Prostheti* OR Osteopath* OR Paramedic* OR Pedorthist OR Perfusion* OR Pharmac* OR Physiotherap* OR Podiatr* OR Psycholog* OR Rehabilitation counsel* OR Social work* OR Sonograph* OR Speech therap* OR Speech language therap* | 6,275 |
| 2 | Tele* OR Phone OR Video OR Remote OR Simulat* | 407 |
| 3 | Competenc* OR Standard* OR Knowledge OR Skill* OR Behavio* | 4,959 |
| 4 | Teach* OR Learn* OR Curriculum OR Prac* OR Course* OR Prog* OR Module OR Apprenticeship OR Professional development | 4,722 |
| 5 | ID1 AND ID2 AND ID3 AND ID4 | 209 |

Date limiter (2012) on ID 5

*Database*: EMBASE

*Date*: 15/07/2022

| **ID** | **Search Term(s)** | **Hits** |
| --- | --- | --- |
| 1 | Art therap* OR Audiolog* OR Biomedical scien* OR Chiropract* OR Chinese medic* OR Clinical scien* OR Diabetes educat* OR Dietetics OR Dietitian OR Diversional therap* OR Drama therap* OR Exercise scien* OR Exercise physio* OR Genetic counsel* OR Hearing aid dispens* OR Radiation oncology medical physics OR Radiation therap* OR Radiograph* OR Music therap* OR Occupational therap* OR Operating department pract* OR Optometr* OR Orthopti* OR Orthoti* OR Prostheti* OR Osteopath* OR Paramedic* OR Pedorthist OR Perfusion* OR Pharmac* OR Physiotherap* OR Podiatr* OR Psycholog* OR Rehabilitation counsel* OR Social work* OR Sonograph* OR Speech therap* OR Speech language therap* | 6,696,108 |
| 2 | Tele* OR Phone OR Video OR Remote OR Simulat* | 1,366,513 |
| 3 | Competenc* OR Standard* OR Knowledge OR Skill* OR Behavio* | 5,741,753 |
| 4 | Teach* OR Learn* OR Curriculum OR Prac* OR Course* OR Prog* OR Module OR Apprenticeship OR Professional development | 8,545,987 |
| 5 | ID1 AND ID2 AND ID3 AND ID4 | 2105 |

Date limiter (2012) on ID 5

*Database*: Web of Science

*Date*: 15/07/2022

| **ID** | **Search Term(s)** | **Hits** |
| --- | --- | --- |
| 1 | Art therap* OR Audiolog* OR Biomedical scien* OR Chiropract* OR Chinese medic* OR Clinical scien* OR Diabetes educat* OR Dietetics OR Dietitian OR Diversional therap* OR Drama therap* OR Exercise scien* OR Exercise physio* OR Genetic counsel* OR Hearing aid dispens* OR Radiation oncology medical physics OR Radiation therap* OR Radiograph* OR Music therap* OR Occupational therap* OR Operating department pract* OR Optometr* OR Orthopti* OR Orthoti* OR Prostheti* OR Osteopath* OR Paramedic* OR Pedorthist OR Perfusion* OR Pharmac* OR Physiotherap* OR Podiatr* OR Psycholog* OR Rehabilitation counsel* OR Social work* OR Sonograph* OR Speech therap* OR Speech language therap* | 3,132,302 |
| 2 | Competenc* OR Standard* OR Knowledge OR Skill* OR Behavio* | 8,802,835 |
| 3 | Teach* OR Learn* OR Curriculum OR Prac* OR Course* OR Prog* OR Module OR Apprenticeship OR Professional development | 9,874,203 |
| 4 | ID1 AND ID2 AND ID3 AND Tele* | 5,765 |
| 5 | ID1 AND ID2 AND ID3 AND Phone | 1,678 |
| 6 | ID1 AND ID2 AND ID3 AND Video | 4,287 |
| 7 | ID1 AND ID2 AND ID3 AND Remote | 1,887 |
| 8 | ID1 AND ID2 AND ID3 AND Simulat* | 7,271 |

Date limiter (2012) from ID 4 onwards

*Database*: PEDro

*Date*: 22/07/2022

| **ID** | **Search Term(s)** | **Hits** |
| --- | --- | --- |
| 1 | Tele* AND Competen* | 4 |
| 2 | Tele* AND Standard* | 181 |
| 3 | Tele* AND Knowledge | 36 |
| 4 | Tele* AND Skill* | 46 |
| 5 | Tele* AND Behavio* | 263 |

Date limiter (2012) for all

*Database*: WHO

*Date*: 22/07/2022

| **ID** | **Search Term(s)** | **Hits** |
| --- | --- | --- |
| 1 | Telehealth | 0 |

No date limiter

*Database*: Health Education England

*Date*: 22/07/2022

| **ID** | **Search Term(s)** | **Hits** |
| --- | --- | --- |
| 1 | Telehealth competence | 283 |

No date limiter

Only the first 100 hits were scanned for all searches

**Searches on Professional Body Websites**

*Database*: Biomedical scientists – Institute of biomedical scientists

*Date*: 8.8.22

| **ID** | **Search Term(s)** | **Hits** |
| --- | --- | --- |
| 1 | Telehealth and competence | 1 |
| 2 | Telehealth and standard | 3 |
| 3 | Tele and knowledge | 3 |
| 4 | Tele and skill | 2 |
| 5 | Tele and behaviour | 2 |

No date limiter

*Database*: Podiatrists – Royal College of Podiatry

*Date*: 8.8.22

| **ID** | **Search Term(s)** | **Hits** |
| --- | --- | --- |
| 1 | Telehealth and competence | 12 |
| 2 | Telehealth and standard | 12 |
| 3 | Tele and knowledge | 13 |
| 4 | Tele and skill | 12 |
| 5 | Tele and behaviour | 12 |

No date limiter

*Database*: Clinical scientists – Professional Bodies Council (Academy for Healthcare Science is the over-arching professional body for Healthcare Science)

*Date*: 8.822

| **ID** | **Search Term(s)** | **Hits** |
| --- | --- | --- |
| 1 | Telehealth and competence | 0 |
| 2 | Telehealth and standard | 0 |
| 3 | Tele and knowledge | 0 |
| 4 | Tele and skill | 0 |
| 5 | Tele and behaviour | 0 |

No date limiter

*Database*: Osteopaths – General Osteopath Council

*Date*: 8.822

| **ID** | **Search Term(s)** | **Hits** |
| --- | --- | --- |
| 1 | Telehealth | 10 |
| 2 | Telehealth and competence | 0 |
| 3 | Telehealth and standard | 0 |
| 4 | Tele and knowledge | 0 |
| 5 | Tele and skill | 0 |
| 6 | Tele and behaviour | 0 |

No date limiter

*Database*: Osteopaths – Institute of Osteopathy

*Date*: 8.822

| **ID** | **Search Term(s)** | **Hits** |
| --- | --- | --- |
| 1 | Telehealth | 4 |
| 2 | Telehealth and competence | 0 |
| 3 | Telehealth and standard | 0 |
| 4 | Tele and knowledge | 0 |
| 5 | Tele and skill | 0 |
| 6 | Tele and behaviour | 0 |

No date limiter

*Database*: British Association of Prosthetists and Orthotists (BAPO)

*Date*: 8.822

| **ID** | **Search Term(s)** | **Hits** |
| --- | --- | --- |
| 1 | Telehealth | 1 |
| 2 | Telehealth and competence | 0 |
| 3 | Telehealth and standard | 0 |
| 4 | Tele and knowledge | 0 |
| 5 | Tele and skill | 0 |
| 6 | Tele and behaviour | 0 |

No date limiter

*Database*: Institute for Arts in Therapy and Education

*Date*: 8.8.22

| **ID** | **Search Term(s)** | **Hits** |
| --- | --- | --- |
| 1 | Tele* | 0 |
| 2 | Telehealth | 0 |
| 3 | Telerehabilitation | 0 |
| 4 | Competen* | 0 |
| 5 | Competencies | 0 |
| 6 | Competence | 8 |
| 7 | Standard | 7 |
| 8 | Knowledge | 10 |
| 9 | Skill | 1 |
| 10 | Behaviour | 8 |

No date limiter

*Database:* Royal College of Occupational Therapists

*Date*: 8.8.22

| **ID** | **Search Term(s)** | **Hits** |
| --- | --- | --- |
| 1 | Tele* | 7 |
| 3 | Telehealth | 0 |
| 2 | Telerehabilitation | 0 |
| 4 | Competence AND telehealth | 2 |
| 5 | Standard AND telehealth | 2 |
| 6 | Knowledge AND telehealth | 2 |
| 7 | Skill AND teleheatlh | 3 |
| 8 | Behaviour AND telehealth | 0 |

Date limiter (2012) for all

*Database*: Chartered Society of Physiotherapy

*Date*: 9.8.22

| **ID** | **Search Term(s)** | **Hits** |
| --- | --- | --- |
| 1 | Tele* | 0 |
| 2 | Telehealth | 96 |
| 3 | Telerehabilitation | 12 |
| 4 | Competence | 309 |
| 5 | Telehealth AND competence | 22442 |
| 6 | Standard | 1218 |
| 7 | Standard AND telehealth | 22475 |
| 8 | Knowledge AND telehealth | 22448 |
| 9 | Knowledge | 2260 |
| 10 | Skill AND telehealth | 22480 |
| 11 | Skill | 2698 |
| 12 | Behaviour AND telehealth | 22441 |
| 13 | Behaviour | 505 |

Date limiter (2012) for all

*Database*: Australian Physiotherapy Association

*Date*: 9.8.22

| **ID** | **Search Term(s)** | **Hits** |
| --- | --- | --- |
| 1 | Telehealth AND competence | 5 |
| 2 | telerehabilitation | 0 |
| 3 | Standard AND telehealth | 21 |
| 4 | Knowledge AND telehealth | 35 |
| 5 | Skill AND telehealth | 9 |
| 6 | Behaviour AND telehealth | 15 |

Date limiter (2012) for all

*Database*: Exercise and Sports Science Australia

*Date*: 9.8.22

| **ID** | **Search Term(s)** | **Hits** |
| --- | --- | --- |
| 1 | Tele* | 27 |
| 2 | Telehealth |  |
| 3 | Telerehabilitation | 0 |
| 4 | Competence | 20 |
| 5 | Standard | 93 |
| 6 | Knowledge | 125 |
| 7 | Skill | 102 |
| 8 | Behaviour | 40 |

Date limiter (2012) for all

*Database*: The Association of UK Dietitians

*Date*: 23/08/2022

| **ID** | **Search Term(s)** | **Hits** |
| --- | --- | --- |
| 1 | Telehealth | 10 |
| 2 | Tele | 1 |
| 3 | Digital health | 12 |
| 4 | Competence | 85 |

No date limiter

*Database*: Dietitians Australia

*Date*: 23/08/2022

| **ID** | **Search Term(s)** | **Hits** |
| --- | --- | --- |
| 1 | Telehealth | 26 |
| 2 | Tele | 0 |
| 3 | Competence | 30 |

No date limiter

*Database*: The British Association of Drama Therapists

*Date*: 23/08/2022

| **ID** | **Search Term(s)** | **Hits** |
| --- | --- | --- |
| 1 | Telehealth | 0 |
| 2 | Tele | 0 |
| 3 | Competence | 0 |
| 4 | Standard | 0 |

No date limiter

*Database*: British Society of Hearing Aid Audiologists

*Date*: 23/08/2022

| **ID** | **Search Term(s)** | **Hits** |
| --- | --- | --- |
| N/A | UNABLE TO SEARCH (No search function for public)  Instead, manually explored site | N/A |

No date limiter

*Database*: Audiology Australia

*Date*: 23/08/2022

| **ID** | **Search Term(s)** | **Hits** |
| --- | --- | --- |
| 1 | Telehealth | 11 |
| 2 | Tele | 13 |
| 3 | Competence | 2 |

No date limiter

*Database*: British Association for Music Therapy

*Date*: 23/08/2022

| **ID** | **Search Term(s)** | **Hits** |
| --- | --- | --- |
| N/A | UNABLE TO SEARCH (No search function for public)  Instead, manually explored site | N/A |

No date limiter

*Database*: Australian Music Therapy Association

*Date*: 23/08/2022

| **ID** | **Search Term(s)** | **Hits** |
| --- | --- | --- |
| 1 | Telehealth | 12 |
| 2 | Tele | 1 |
| 3 | Competence | 2 |
| 4 | Standard | 0 |

No date limiter

*Database*: College of Operating Department Practitioners

*Date*: 23/08/2022

| **ID** | **Search Term(s)** | **Hits** |
| --- | --- | --- |
| 1 | Telehealth | 0 |
| 2 | Tele | 466 |
| 3 | Competence | 616 |

No date limiter

*Database*: The British and Irish Orthoptic Society

*Date*: 23/08/2022

| **ID** | **Search Term(s)** | **Hits** |
| --- | --- | --- |
| 1 | Telehealth | 0 |
| 2 | Tele | 22 |
| 3 | Competence | 0 |

No date limiter

*Database*: Orthoptics Australia

*Date*: 23/08/2022

| **ID** | **Search Term(s)** | **Hits** |
| --- | --- | --- |
| 1 | Telehealth | 6 |
| 2 | Tele | 0 |
| 3 | Competence | 0 |
| 4 | Digital health | 10 |

No date limiter

*Database*: The College of Paramedics

*Date*: 23/08/2022

| **ID** | **Search Term(s)** | **Hits** |
| --- | --- | --- |
| 1 | Telehealth | 1 |
| 2 | Tele | 0 |
| 3 | Competence | 29 |
| 4 | Digital health | 10 |

No date limiter

*Database*: The Australasian College of Paramedicine

*Date*: 23/08/2022

| **ID** | **Search Term(s)** | **Hits** |
| --- | --- | --- |
| N/A | UNABLE TO SEARCH (No search function for public)  Instead, manually explored site | N/A |

No date limiter

*Database*: The British Psychological Society

*Date*: 23/08/2022

| **ID** | **Search Term(s)** | **Hits** |
| --- | --- | --- |
| 1 | Telehealth | 12 |
| 2 | Tele | 45 |
| 3 | Digital health | 895 |

No date limiter

*Database*: The Australian Clinical Psychology Association

*Date*: 23/08/2022

| **ID** | **Search Term(s)** | **Hits** |
| --- | --- | --- |
| 1 | Telehealth | 2 |
| 2 | Tele | 0 |
| 3 | Digital health | 0 |
| 4 | Competence | 0 |
| 5 | Standard | 1 |

No date limiter

*Database*: The Society of Radiographers

*Date*: 23/08/2022

| **ID** | **Search Term(s)** | **Hits** |
| --- | --- | --- |
| 1 | Telehealth | 0 |
| 2 | Tele | 3 |
| 3 | Digital health | 2279 |

No date limiter

*Database*: The Royal College of Speech and Language Therapists

*Date*: 23/08/2022

| **ID** | **Search Term(s)** | **Hits** |
| --- | --- | --- |
| 1 | Telehealth | 0 |
| 2 | Tele | 4 |
| 3 | Digital health | 1 |

No date limiter

*Database*: Speech Pathology Australia

*Date*: 23/08/2022

| **ID** | **Search Term(s)** | **Hits** |
| --- | --- | --- |
| 1 | Telehealth | 14 |
| 2 | Tele | 5 |
| 3 | Digital health | 16 |

No date limiter

*Database*: ANZACATA

*Date*: 25/08/2022

| **ID** | **Search Term(s)** | **Hits** |
| --- | --- | --- |
| 1 | Telehealth | 0 |
| 2 | Tele | 0 |
| 3 | Digital health | 17 |
| 4 | Standards | 9 |
| 5 | Competence | 2 |

No date limiter

*Database*: The Australian Chiropractors Association

*Date*: 25/08/2022

| **ID** | **Search Term(s)** | **Hits** |
| --- | --- | --- |
| 1 | Telehealth | 4 |
| 2 | Tele | 15 |
| 3 | Digital health | 12 |
| 4 | Competence | 14 |

No date limiter

*Database*: The Australian Acupuncture and Chinese Medicine Association

*Date*: 25/08/2022

| **ID** | **Search Term(s)** | **Hits** |
| --- | --- | --- |
| 1 | Telehealth | 1 |
| 2 | Tele | 3 |
| 3 | Digital health | 0 |
| 4 | Competence | 0 |
| 5 | Standard | 8 |

No date limiter

*Database*: The Australian Diabetes Educators Association

*Date*: 25/08/2022

| **ID** | **Search Term(s)** | **Hits** |
| --- | --- | --- |
| 1 | Telehealth | 6 |
| 2 | Tele | 12 |
| 3 | Digital health | 273 |
| 4 | Competence | 5 |

No date limiter

*Database*: The Diversional and Recreation Therapy Australia

*Date*: 25/08/2022

| **ID** | **Search Term(s)** | **Hits** |
| --- | --- | --- |
| 1 | Telehealth | 12 |
| 2 | Tele | 12 |
| 3 | Digital health | 12 |

No date limiter

*Database*: The Human Genetics Society of Australasia

*Date*: 25/08/2022

| **ID** | **Search Term(s)** | **Hits** |
| --- | --- | --- |
| 1 | Telehealth | 0 |
| 2 | Tele | 8 |
| 3 | Digital health | 1 |
| 4 | Competence | 3 |

No date limiter

*Database*: The Australian Society of Medical Imaging and Radiation Therapy

*Date*: 25/08/2022

| **ID** | **Search Term(s)** | **Hits** |
| --- | --- | --- |
| 1 | Telehealth | 3 |
| 2 | Tele | 8 |
| 3 | Digital health | 15 |
| 4 | Competence | 2 |

No date limiter

*Database*: Occupational Therapy Australia

*Date*: 25/08/2022

| **ID** | **Search Term(s)** | **Hits** |
| --- | --- | --- |
| 1 | Telehealth | 44 |
| 2 | Tele | 45 |
| 3 | Digital health | 23 |

No date limiter

*Database*: Optometry Australia

*Date*: 25/08/2022

| **ID** | **Search Term(s)** | **Hits** |
| --- | --- | --- |
| 1 | Telehealth | 50 |
| 2 | Tele | 50 |
| 3 | Digital health | 50 |

No date limiter

*Database*: The Australian Orthotic Prosthetic Association

*Date*: 25/08/2022

| **ID** | **Search Term(s)** | **Hits** |
| --- | --- | --- |
| N/A | UNABLE TO SEARCH (No search function for public)  Instead, manually explored site | N/A |

No date limiter

*Database*: Osteopathy Australia

*Date*: 25/08/2022

| **ID** | **Search Term(s)** | **Hits** |
| --- | --- | --- |
| 1 | Telehealth | 30 |
| 2 | Tele | 38 |
| 3 | Digital health | 13 |

No date limiter

*Database*: Pedorthic Association of Australia

*Date*: 25/08/2022

| **ID** | **Search Term(s)** | **Hits** |
| --- | --- | --- |
| N/A | UNABLE TO SEARCH (No search function for public)  Instead, manually explored site | N/A |

No date limiter

*Database*: The Australian and New Zealand College of Perfusionists

*Date*: 25/08/2022

| **ID** | **Search Term(s)** | **Hits** |
| --- | --- | --- |
| 1 | Telehealth | 0 |
| 2 | Tele | 7 |
| 3 | Digital health | 0 |
| 4 | Competence | 2 |

No date limiter

*Database*: The Society of Hospital Pharmacists of Australia

*Date*: 25/08/2022

| **ID** | **Search Term(s)** | **Hits** |
| --- | --- | --- |
| 1 | Telehealth | 2 |
| 2 | Tele | 8 |
| 3 | Digital health | 5 |

No date limiter

*Database*: General Pharmaceutical Council

*Date*: 25/08/2022

| **ID** | **Search Term(s)** | **Hits** |
| --- | --- | --- |
| 1 | Telehealth | 6 |
| 2 | Tele | 455 |
| 3 | Digital health | 62 |
| 4 | Competence | 452 |

No date limiter

*Database*: Australian Podiatry Association

*Date*: 25/08/2022

| **ID** | **Search Term(s)** | **Hits** |
| --- | --- | --- |
| 1 | Telehealth | 25 |
| 2 | Tele | 65 |
| 3 | Digital health | 13 |
| 4 | Competence | 4 |

No date limiter

*Database*: The Rehabilitation Counselling Association of Australasia

*Date*: 25/08/2022

| **ID** | **Search Term(s)** | **Hits** |
| --- | --- | --- |
| 1 | Telehealth | 0 |
| 2 | Tele | 0 |
| 3 | Digital health | 19 |
| 4 | Competence | 41 |

No date limiter

*Database*: The Professional Association for Social Work and Social Workers

*Date*: 25/08/2022

| **ID** | **Search Term(s)** | **Hits** |
| --- | --- | --- |
| 1 | Telehealth | 6 |
| 2 | Tele | 3 |
| 3 | Digital health | 6597 |

No date limiter

*Database*: The Australian Association of Social Workers

*Date*: 25/08/2022

| **ID** | **Search Term(s)** | **Hits** |
| --- | --- | --- |
| 1 | Telehealth | 21 |
| 2 | Tele | 47 |
| 3 | Digital health | 20 |

No date limiter

*Database*: Australasian Sonographers Association

*Date*: 25/08/2022

| **ID** | **Search Term(s)** | **Hits** |
| --- | --- | --- |
| 1 | Telehealth | 1 |
| 2 | Tele | 1 |
| 3 | Digital health | 1 |

No date limiter
